# Supplementary material for: Splice-Junction-Based Mapping of Alternative Isoforms in the Human Proteome
Source: Cell Rep. Author manuscript; Available in PMC 2020 Jan 15. (PMC6961840; doi:10.1016/j.celrep.2019.11.026)
Supplement: 2 [file NIHMS1546469-supplement-2.zip › DF1/PXD009737/TestisLysC/TestisLysC_11_UGGT1_KDDSFIRPVTFWIVGDFDSPSGRQLLYDAIK.pdf]

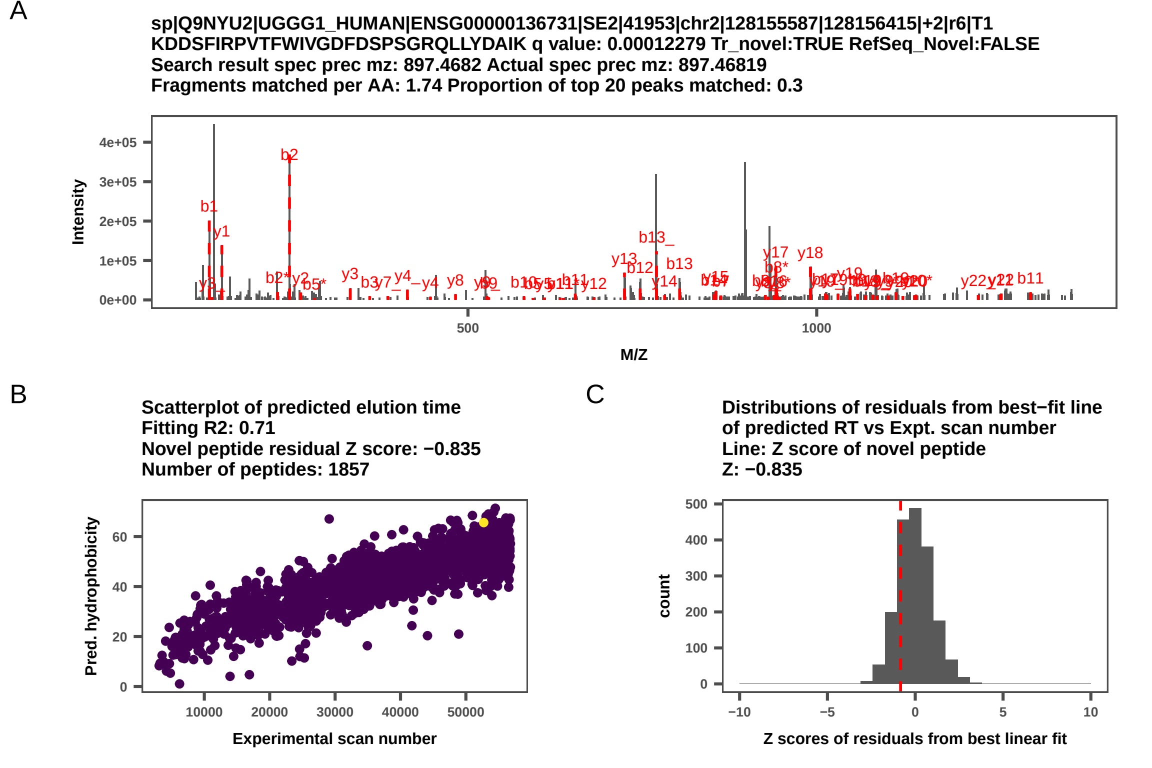
A
sp|Q9NYU2|UGGG1_HUMAN|ENSG00000136731|SE2|41953|chr2|128155587|128156415|+2|r6|T1
KDDSFIRPVTFWIVGDFDSPSGRQLLYDAIK q value: 0.00012279 Tr_novel:TRUE RefSeq_Novel:FALSE
Search result spec prec mz: 897.4682 Actual spec prec mz: 897.46819
Fragments matched per AA: 1.74 Proportion of top 20 peaks matched: 0.3
Intensity
4e+05
3e+05
2e+05
1e+05
0e+00
500
1000
M/Z
b1
y1
b2
y3
y13
b12
b13
b13_
y14
y17
y18
y22
b11
B
Scatterplot of predicted elution time
Fitting R2: 0.71
Novel peptide residual Z score: −0.835
Number of peptides: 1857
Pred. hydrophobicity
60
40
20
0
10000
20000
30000
40000
50000
Experimental scan number
C
Distributions of residuals from best−fit line
of predicted RT vs Expt. scan number
Line: Z score of novel peptide
Z: −0.835
count
500
400
300
200
100
0
−10
−5
0
5
10
Z scores of residuals from best linear fit
